# Protective effect of cocoa antioxidant extract in distinct vehicles on dentin erosion *in vitro*

**DOI:** 10.1590/1807-3107bor-2026.vol40.038

**Published:** 2026-06-22

**Authors:** Gabriela Carvalho Santos Fernandes, Maria Eduarda Martins Bentes, Tayanne Laise da Rocha Pirixan Louzeiro, Pedro Henrique Campos Pimentel, Gilson Celso Albuquerque Chagas-Junior, Nelson Rosa Ferreira, Maria do Perpétuo Socorro Progene Vilhena, Daniela Pinheiro Gaspar, Cristiane de Melo Alencar

**Affiliations:** (a) Centro Universitário do Estado do Pará – Cesupa, School of Dentistry, Dental Clinic Department, Belém, PA, Brazil.; (b) Universidade Federal do Pará – UFPA, Institute of Technology, Laboratory of Biotechnological Processes, Graduate Program in Food Science and Technology (PPGCTA), Belém, PA, Brazil.; (c) Universidade Federal Rural da Amazônia – UFRA, Socio-Environmental and Water Resources Institute, Belém, PA, Brazil.

**Keywords:** Antioxidants, Dentin, Erosion, Tooth Erosion

## Abstract

This study aimed to conduct an *in vitro* evaluation of the antierosive potential of a solution and an experimental toothpaste enriched with natural antioxidants derived from cocoa (*Theobroma cacao L.)* on eroded dentin. Cocoa beans underwent fermentation, drying, roasting, grinding, and freeze-drying to obtain an aqueous cocoa extract. The extract was analyzed for total polyphenols and antioxidant capacity using the oxygen radical antioxidant capacity (ORAC) assay. A control toothpaste was then formulated and enriched with the concentrated cocoa extract. Eighty bovine dentin specimens were pre-eroded in citric acid and randomized into four groups (n = 20): G1 (negative control) – toothpaste without active ingredients; G2 – concentrated cocoa polyphenol solution; G3 – experimental toothpaste enriched with cocoa polyphenols; and G4 (positive control) – Elmex Protect commercial toothpaste (Colgate). Specimens underwent 3 days of erosive cycling. Surface loss (dSL-eroded) was measured by optical profilometry; collagen degradation (dColl) was calculated after collagenase exposure; and calcium release (CaR) was quantified by atomic absorption spectrometry. Surface morphology was qualitatively assessed by scanning electron microscopy (SEM). Data were analyzed by one-way ANOVA and Tukey's test (α=0.05). The cocoa extract showed higher levels of total polyphenols than did the enriched toothpaste (p = 0.037). Antioxidant activity decreased after toothpaste manipulation (p = 0.025). G2 exhibited significantly lower dSL-eroded, dColl, dSL-total, and calcium release compared with the other groups (p<0.05). SEM analysis of G3 revealed partially or totally occluded dentinal tubules. In conclusion, cocoa extract exhibited a higher polyphenol content and significant antierosive effects, leading to reduced dentin surface loss and enhanced tubule occlusion.

## Introduction

Erosive tooth wear (ETW), defined as the progressive loss of hard tissues by chemical processes without bacterial involvement, is increasingly prevalent across age groups, particularly among adolescents and young adults because of dietary and lifestyle factors. A systematic review reported prevalence rates ranging from 30% to 50% among children and adolescents across different populations.^1^ The pathogenesis of ETW involves enamel and dentin demineralization following exposure to acids at pH levels below the critical threshold for hydroxyapatite dissolution (~5.5). This process is aggravated by intrinsic factors, such as gastric acid associated with gastroesophageal reflux, and extrinsic factors, including the frequent intake of acidic foods and beverages.^1^ Repeated acid exposure softens enamel, leading to surface loss, dentin exposure, and hypersensitivity.^3^

Management strategies focus on prevention, remineralization, and, when indicated, restorative intervention.^4^ Preventive measures include dietary counseling to limit acidic intake and stimulation of salivary flow to enhance buffering capacity. Remineralization commonly relies on fluoride products, which promote the formation of acid-resistant mineral phases,^5^ while casein phosphopeptide-amorphous calcium phosphate (CPP-ACP) has been investigated for its ability to promote mineral gain and inhibit demineralization. In advanced cases, adhesive restorative materials are required to recover function and aesthetics.^6^

Titanium tetrafluoride (TiF_4_) is considered a promising agent for the prevention of ETW owing to its distinct mechanism of action.^7^ When applied, TiF_4_ forms a glaze-like layer composed mainly of titanium dioxide (TiO_2_) and fluoride compounds, increasing enamel resistance to acid. Titanium ions facilitate precipitation of this acid-resistant layer, thereby limiting demineralization under acidic challenge.^8^ Nevertheless, current evidence remains inconclusive because of methodological heterogeneity and low study quality.^9^ Recently, natural antioxidants have also shown potential in preventing ETW.^10,11^ In advanced stages, dentin exposure renders the organic matrix vulnerable to degradation by matrix metalloproteinases (MMPs).^12^ These collagenolytic enzymes are secreted as zymogens and may be activated in acidic environments such as those found in erosive challenges and adhesive procedures.^13^ To mitigate this process, dentin-biomodification strategies, including the use of MMP inhibitors, have been proposed to stabilize the adhesive interface and protect collagen from enzymatic breakdown.^14^ Nonetheless, the most recent clinical studies investigating the longevity of adhesive restorations have suggested MMP inhibition appears to have no significant effect on restoration retention or survival.^15^

*Theobroma cacao* contains phenolic compounds in its phytochemical composition, which exhibit antioxidant, anti-inflammatory, antimicrobial, anticancer, and other biological properties that underscore the nutritional and therapeutic value of this fruit in human health.^16^ Cocoa seeds contain high levels of polyphenolic compounds, notably chlorogenic acid, protocatechuic acid, epigallocatechin, gallocatechin, epicatechin, and procyanidins. Additionally, methylxanthines such as caffeine, theobromine, and theophylline are present in cocoa beans.^16,17^ The strong antioxidant activity of cocoa polyphenols has sparked significant scientific and pharmacological interest in dentistry for the development of novel dental materials derived from cocoa seeds.

Notwithstanding the recognized antioxidant potential of cocoa polyphenols, evidence regarding their ability to prevent dentin erosion and the influence of different formulation vehicles on their antierosive performance remains limited. This knowledge gap underpinned the present study, which evaluated *in vitro* the antierosive potential of a solution and an experimental toothpaste enriched with natural antioxidants derived from cocoa (*Theobroma cacao L*.) on eroded dentin. The following null hypotheses were tested: (H01) the use of a solution containing antioxidant extract from *Theobroma cacao* L. has no effect on the control of eroded dentin; and (H02) the use of a toothpaste containing antioxidant extract from *Theobroma cacao* L. has no effect on the control of eroded dentin.

## Methods

An a priori power analysis (G*Power 3.1; one-way ANOVA, four groups; α = 0.05; power = 0.80) was performed based on pilot data for the primary outcome (dentin surface loss, μm; SD ≈ 16–20 μm). The analysis indicated that a minimum of 72 specimens (f ≈ 0.35; η^2^ ≈ 0.11) would be required to detect clinically relevant between-group differences. Accordingly, 20 specimens per group (n = 80) were included to ensure adequate power and account for potential specimen loss.

Bovine incisors from recently slaughtered cattle were used, with ethical approval waived by the Institutional Animal Care and Use Committee. One hundred teeth were sectioned 1 mm apical to the cementoenamel junction using a precision saw (Minitom, Struers, Ballerup, Denmark) at 350 rpm. Cervical root dentin blocks (4 × 4 × 2 mm) were obtained from buccal surfaces, measured with a caliper, and polished with SiC papers (#600, #1200; 3M, St. Paul, USA) under water cooling. Specimens were cleaned in an ultrasonic bath (Cristófoli, Brazil, 3 min) and baseline profiles were recorded using a 3D non-contact profilometer (Nanovea PS50, Nanovea, Irvine, USA). Half of each surface was protected with nail varnish (Risqué, São Paulo, Brazil). Twenty blocks were excluded owing to anomalous baseline profiles; 80 were randomized into four groups (n = 20), with no baseline roughness differences among groups (ANOVA, p > 0.05). Specimens were randomly assigned to the four experimental groups using a computer-generated randomization list (Microsoft Excel). All profilometric measurements and subsequent statistical analyses were performed by a single calibrated examiner who was blinded to the group assignments.

Floodplain cacao fruits were harvested in March 2023 in Mocajuba, Pará, Brazil (02°35′2″ S, 49°30′25″ W). Beans underwent fermentation, drying, roasting, and grinding,^18^ then pulped, frozen at −18°C for 72 h, and lyophilized Lyophilized (Liotop L101, Liotop, São Carlos, Brazil; −54 °C for 72 h). Lyophilized powder was vacuum sealed and stored at −19 °C. Seeds were ground (Ika A11B) and defatted using n-hexane (Neon, São Paulo, Brazil) following Brito et al..^19^ The residue was homogenized in 50% ethanol, sonicated for 30 min (25–40°C; Solid Steel), and centrifuged at 4000 rpm for 15 min.^20^ Supernatant solvent was removed by evaporation in a Dubnoff water bath (60°C, 45 min over 48 h), yielding 45 mL of aqueous extract (35 mL stored in glass bottles; 10 mL reserved for polyphenol analysis). The extract was stored in amber glass bottles at 4°C, protected from light and humidity, and used within 30 days after preparation to minimize oxidation or polyphenol degradation. The experimental toothpaste was prepared immediately before the erosive challenges and kept under the same controlled conditions throughout the study period.

Total phenolic content (TPC) was measured using the Folin–Ciocalteu method and a UV-Vis spectrophotometer (SECOMAM, UV Line 9400, Alès, France) at a wavelength of 750 nm.^21^ The results were expressed as milligrams of gallic acid equivalents (GAE) per gram of sample, based on a gallic acid calibration curve.

The oxygen radical absorbance (ORAC) evaluation was conducted using the ORAC Assay Kit (ab233473) obtained from Abcam (Cambridge, UK), following the protocol described in a previous study.^22^ The absorbance of both sample and standard wells was measured immediately at 37°C using a BioTek Cytation 5 multimode cell imaging reader (Agilent, Santa Clara, USA) with excitation and emission wavelengths set at 480 nm and 520 nm, respectively.

A base toothpaste without an active ingredient was formulated with the following components: carboxymethylcellulose (CMC), glycerin, methylparaben, sorbitol, abrasive silica, titanium dioxide, cocamidopropyl betaine, and water q.s. (compounded at the UNESP pharmacy). The base toothpaste for the experimental group was diluted/enriched to form a slurry (1 part toothpaste to 3 parts cocoa solution). In the negative and positive control groups, the toothpastes were diluted to form a slurry (1 part toothpaste to 3 parts deionized water, w/w) for treatment. The pH of the mixtures was measured in triplicate using a pH meter, previously calibrated with pH standards of 4.1 and 7.0 (Orion 3-Star pH Bench Top, Thermo Electron Corporation, Waltham, USA). In addition, the base toothpaste enriched with cocoa antioxidant extract was also subjected to antioxidant potential analysis using ORAC in the same way as the initial extract.

Initial erosive lesions were induced with 1% citric acid (pH 2.6) for 10 min in 24-well acrylic plates, with one specimen per well.^23^ Specimens were rinsed with distilled water for 10 s and dried with absorbent paper. Half of the eroded surface was covered with unplasticized polyvinyl chloride tape, leaving a 4 × 2 mm exposure window.^24^ Dentin blocks were randomly assigned to four groups (n = 20): G1 – toothpaste without an active ingredient; G2 – concentrated cocoa polyphenol solution; G3 – experimental toothpaste enriched with cocoa polyphenols; G4 – commercial antierosive toothpaste (Elmex Protect, Colgate, GABA International, Switzerland).

The 3-day erosive cycling protocol^25^ consisted of daily application of the assigned treatment, followed by two erosive challenges using 1% citric acid (pH 2.6) for 10 min. Between challenges, specimens were immersed in artificial saliva for 2 h. Solutions were renewed after each cycle, and specimens were stored at 37 °C. Erosive dentin surface loss (dSL-eroded) was measured by non-contact profilometry (Nanovea PS50, USA), using protected areas as reference. Demineralized collagen was removed with type VII collagenase (100 U/mL, Sigma-Aldrich,) for 96 h at 37 °C under agitation. Total dentin surface loss (dSL-total) was then determined, and collagen degradation (dColl) was calculated as dSL-eroded minus dSL-total.^26^

Calcium release (CaR) was assessed by atomic absorption spectrometry (AAnalyst 400, Perkin-Elmer, USA) after each erosive cycle and normalized to the exposed area, as measured by light microscopy (Leica M420/DFC495, 20×, IM500 software).^27^ Surface morphology was qualitatively examined by scanning electron microscopy (JSM-IT300, JEOL, Japan). Specimens were mounted on stubs, sputter-coated with gold (~10–15 nm), and imaged with secondary electrons at 3.0 kV, 15 mm working distance, 1500× magnification.

Data were evaluated for normality using the Shapiro-Wilk test. Given the normal distribution of the data, parametric tests were applied. One-way ANOVA was used for between-group comparisons of all response variables: total polyphenol analysis, antioxidant potential (ORAC), total dentin surface loss (dSL-total), collagen degradation (dColl), and calcium release to citric acid (CaR). Tukey's post-hoc test was performed. Statistical significance was set at 5%, and the analyses were performed using SPSS software version 13.0 (SPSS).

## Results

The formulations presented slightly acidic to neutral pH levels: toothpaste without an active ingredient (pH = 6.5); concentrated cocoa polyphenol solution (pH = 7.2); experimental toothpaste with cocoa polyphenols (pH = 6.3); and commercial antierosive toothpaste (Elmex Protect, GABA International, Switzerland) (pH = 6.1).

The cocoa extract showed a higher total polyphenol content when compared to the toothpaste enriched with cocoa extract (p = 0.037). Antioxidant activity was significantly reduced after incorporation of the extract into the toothpaste formulation (p = 0.025) (Table 1).

**Table 1:** Mean values of total phenolic content and antioxidant activity (ORAC) for cocoa extract and enriched toothpaste.

Compound	Cocoa extract	
	Total phenolics	ORAC-FL index
	(mg GAE/100 g sample)	
Cocoa extract	324.18 (±0.67)^a^	1.523 ± 0.09^a^
Toothpaste enriched with cocoa extract	47.59 (±0.88)^b^	0.669 ± 0.19^b^

FL: relative fluorescence intensity. Values expressed as mean ± standard deviation; Different lowercase letters in the same column indicate statistical significance (Tukey's test, p ≤ 0.05).

Group G2 (Table 2) showed the lowest values for eroded dentin surface loss, collagen degradation, and total dentin surface loss p< 0.05). No statistical difference was observed between G3 and G4 for any of the evaluated outcomes (p > 0.05).

**Table 2:** Mean values of eroded dentin surface loss (dSL-eroded), collagen degradation (dColl), and total dentin surface loss (dSL-total) assessed by 3D profilometry.

Group	(dSL-eroded)	(dColl)	(dSL-total)
G1	-3.72 (± 0.98)^a^	-2,07 (± 0.31)^a^	-5.79 (± 1.59)^a^
G2	-0.33 (± 0.10)^b^	-0.14 (± 0.03)^b^	-0.47 (± 0.08)^b^
G3	- 1.94 (± 0.57)^c^	- 0.72 (± 0.21)^c^	- 2.66 (± 0.54)^c^
G4	- 1.59 (± 0.76)^c^	- 0.94 (± 0.13)^c^	- 2.53 (± 0.48)^c^

Values expressed as mean ± standard deviation. Different lowercase letters in the same column indicate statistical significance (Tukey's test, p ≤ 0.05).

Table 3 shows that G2 exhibited the lowest calcium release to into citric acid (p < 0.05). No statistical difference was observed between G3 and G4 for any of the investigated outcomes (p = 0.871).

**Table 3:** Mean (M) and standard deviation (± SD) of total calcium release (CaR, μg/mm²) into citric acid after erosive challenge.

Group	(CaR)
G1	-72.41 (± 13.05)^a^
G2	-15.04 (± 08.02)^b^
G3	- 43.23 (± 18.77)^c^
G4	- 38.99 (± 16.98)^c^

Values expressed as mean ± standard deviation; Different lowercase letters in the same column indicate statistical significanc (Tukey's test, p ≤ 0.05).

SEM analysis revealed the negative control group (G1) exhibited the greatest loss of tooth structure, with numerous open dentinal tubules compared with the other groups. In contrast, G2 (cocoa solution) displayed a smoother surface topography, characterized by partially or completely occluded dentinal tubules after treatment (Figure).

**Figure:**
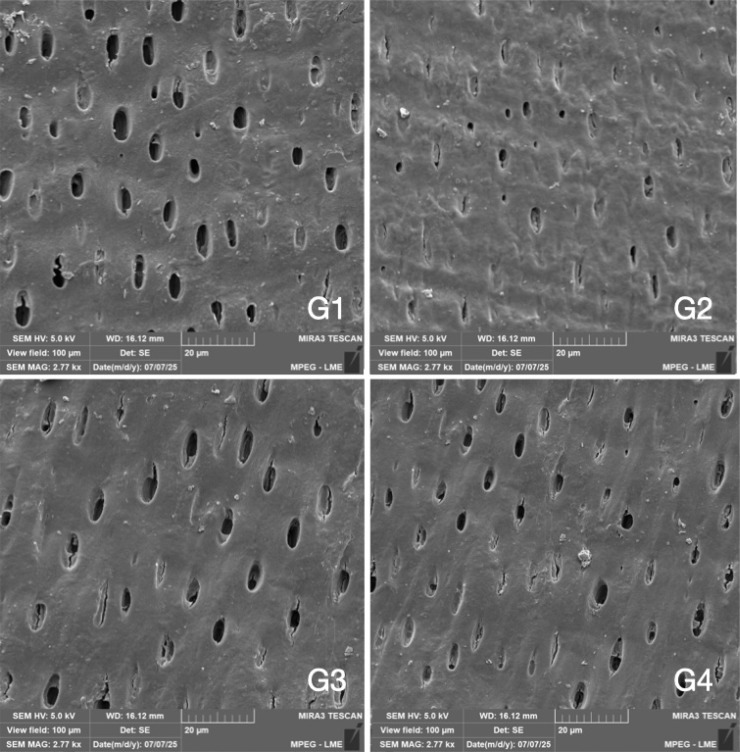
Representative SEM micrographs (×1500) of dentin surfaces after erosive challenge and treatment. G1 – toothpaste without active ingredient, showing open tubules; G2 – cocoa solution with partial tubule occlusion; G3 – toothpaste with cocoa extract, showing smoother surface and narrowed tubules; G4 – commercial antierosive toothpaste with partially open tubules. Scale bar = 10 μm.

## Discussion

Cocoa (*Theobroma cacao*) is widely recognized for its complex chemical composition, particularly its high content of phenolic compounds and strong antioxidant activity. The primary phenolic compounds present in cocoa include flavonoids (e.g., catechins and epicatechins), as well as procyanidins. These compounds are responsible for various health-promoting properties, including potent antioxidant activity, which aids in neutralizing free radicals and preventing cellular damage.^28^ On the other hand, theobromine, the predominant xanthine in *Theobroma cacao*, has already demonstrated promising effects on enamel surface hardness and topography.^29^ Although specific studies on the effects of cocoa extract on eroded dentin are still lacking, a recent systematic review and meta-analysis conducted by da Silva et al.^30^ has shown that *Theobroma cacao* applications can increase the microhardness of demineralized enamel, with results comparable to those of fluoride-based treatments.

The findings of the present study showed that cocoa extract had a higher total polyphenol content when compared with toothpaste enriched with cocoa extract. On the other hand, ORAC analysis revealed a significant decrease in antioxidant activity after toothpaste manipulation, suggesting loss of antioxidant capacity during the handling process. The high polyphenol content in the cocoa solution is in line with research indicating values greater than 300 mg GAE/100 g for minimally processed cocoa derivatives. The content of these compounds, however, can vary significantly depending on factors such as cocoa variety, growing conditions, fermentation processes, drying, and other processing methods.^31^ In addition, the cocoa solution showed potent antioxidant activity, comparable to that of other polyphenol-rich natural compounds. Studies have shown that the antioxidant activity of cocoa extracts vary according to the processing methods and geographic origin of the cocoa.^32,33^ Considering this variability, it has been suggested that formulations designed for oral application should deliver a total polyphenol concentration within the range of approximately 324.18 mg GAE/g and an ORAC-FL index of 1,523. This range may serve as a practical reference for optimizing the biological effectiveness of cocoa-based formulations while maintaining adequate physicochemical properties.

The antioxidant activity of the enriched toothpaste (135.41 μg/mL) was lower than that of the cocoa solution. This finding can be attributed to several factors: a) dilution of antioxidant compounds within the toothpaste matrix, which contains abrasive agents, detergents, and thickeners, potentially reducing the effective concentration of polyphenols available to neutralize free radicals;^34^ and b) degradation of polyphenols during processing and storage as a result of oxidation or interactions with structuring agents, which may hinder the release and bioavailability of antioxidants, making them less reactive in the ORAC assay.^35^

Regarding eroded dentin surface loss, the group treated with the cocoa solution exhibited the lowest values for eroded dentin surface loss, collagen degradation, and total dentin surface loss. No statistical differences were observed between the experimental and commercial toothpaste-treated groups in any of the investigated dental conditions. Accordingly, H01 was rejected and H02 was accepted. Dental erosion occurs when prolonged exposure to acids from dietary, gastric, or bacterial origin disrupts the pH balance of the tooth surface and saliva, reducing salivary buffering capacity. This imbalance leads to the loss of calcium (Ca^2+^) and phosphate (PO_4_^3−^) ions from hydroxyapatite crystals, rendering the dentin more vulnerable and susceptible to erosive challenges than enamel.^11^ Furthermore, acidic challenges promote the release of enzymes such as MMPs and cathepsin K, which degrade the organic collagen matrix. This enzymatic activity softens the tooth surface, accelerating mineral dissolution, increasing surface roughness, and causing loss of dentin volume. As a result, superficial lesions may develop in the dental tissue.^12^

Thus, controlling both MMP and acid challenge effects on the tooth structure is essential to prevent the progression of collagen organic matrix degradation and further mineral loss. In the present study, a cocoa-derived antioxidant solution demonstrated significant effectiveness in preventing ETW. The protective effect observed can be attributed to the capacity of natural antioxidants to prevent and control dentin erosion by neutralizing reactive oxygen species (ROS) and inhibiting enzymes that degrade the organic matrix.^36^ A study by Zanatta et al. ^37^ specifically investigated the inhibitory effects of antioxidants such as polyphenols and flavonoids on MMPs, enzymes responsible for collagen matrix degradation in dentin. By blocking the action of MMPs, not only did natural antioxidants reduce dentin surface softening but they also prevented the increase in surface roughness and tissue volume loss, factors that contribute the progression of erosive lesions.

Furthermore, a study by Buzalaf & Pessan^38^ highlights that many natural antioxidants, such as those found in green tea and citrus fruit extracts, have anti-inflammatory and remineralizing properties that may complement their antierosive effects. These compounds work synergistically to protect dentin by reducing mineral dissolution and strengthening the tooth structure.^38^ Another possible explanation for the antierosive effect observed in the group treated with the cocoa antioxidant solution could be the presence of theobromine, a chemical compound found predominantly in cocoa beans. Theobromine primarily acts through two interconnected mechanisms: stimulation of remineralization and suppression of demineralization in dental tissues. Studies have shown that theobromine facilitates the incorporation of calcium and phosphate ions into the tooth structure, thereby enhancing the stability of hydroxyapatite, the primary mineral component of enamel and dentin.^39^ The potential remineralizing effect of cocoa polyphenols may be hypothesized based on their reported capacity to promote mineral deposition and reduce demineralization, which may help explain the lower calcium ion loss observed in the group treated with the cocoa solution.

Fluoride-based agents and remineralizing compounds such as CPP-ACP and TiF_4_ are well established for their antierosive properties.^6–8^ Their protective effects are primarily attributed to the formation of acid-resistant surface complexes and the delivery of bioavailable calcium and phosphate ions, which promote remineralization and decrease enamel and dentin solubility. In contrast, the cocoa extract evaluated in this study appears to act through a distinct biological pathway. Its polyphenols and methylxanthines exhibit antioxidant activity and may contribute to the preservation of the organic matrix by mitigating oxidative stress and limiting the enzymatic degradation associated with erosive challenges.^39^ To date, no studies have directly compared cocoa-based formulations with conventional antierosive agents, reinforcing the novelty of the present findings and suggesting a promising avenue for future research.

In parallel, *Theobroma cacao* naturally contains theobromine, a methylxanthine compound that has been reported in the literature to promote the formation of a protective layer on the tooth surface, thereby reducing the solubility of enamel and dentin in acidic environments.^39^ This mechanism may help explain, at least in part, the scanning electron microscopy findings in the present study, which demonstrated partial or total obliteration of dentinal tubules in the groups treated with cocoa extract, either in solution or incorporated into toothpaste. Although theobromine was not quantified in the present study, its possible contribution to the observed effects is hypothesized based on its well-documented high concentration in cocoa beans,^38^ which served as the source material in this study. Therefore, the quantification of theobromine is highly encouraged in future investigations and represents a limitation of the present study.

Saliva plays a central role in demineralization and remineralization; however, physiological changes in pH can reduce the availability of free phosphate and hydroxide ions, increasing apatite solubility and hindering the formation of a new hydroxyapatite layer. This underscores the importance of using remineralizing dentifrices to mitigate the effects of acid challenges.^32^ In this study, the use of artificial saliva may have attenuated the biomodifying effects of the salivary pellicle on the antierosive process. This represents a limitation when extrapolating our *in vitro* findings to clinical scenarios.

This study represents an initial and innovative effort to explore *Theobroma cacao* extract as a natural antierosive agent. Although the absence of an inert control group (e.g., distilled water) prevents us from attributing the observed effects solely to the active compound, the experimental design allowed us to evaluate the behavior of the cocoa extract both in solution and incorporated into a toothpaste formulation. These preliminary findings provide encouraging evidence that cocoa-derived polyphenols may help protect against dentin surface loss, supporting further investigation into the underlying mechanisms and the optimization of formulations in future studies to preserve antioxidant stability — for example, through microencapsulation or the use of airtight, opaque packaging.

From a translational perspective, the development of natural and affordable antierosive formulations may be particularly relevant for populations with limited access to dental care. Epidemiological evidence indicates that ETW is often more prevalent in socioeconomically disadvantaged groups, in which preventive measures and restorative treatments are less accessible.^40^ In this context, cocoa-based bioactive formulations could represent a sustainable and cost-effective strategy to mitigate dentin erosion, contributing to oral health promotion and supporting the use of local natural resources.

## Conclusion

This study demonstrated that cocoa extract has a higher polyphenol content compared with the enriched toothpaste, and that the experimental solution exhibited notable antierosive effects by reducing dentin wear and promoting partial obliteration of dentinal tubules.

## Data Availability

The datasets generated during and/or analyzed during the current study are available from the corresponding author on reasonable request.
